# Physicians’ Mutual Benefit Ass’n of Texas

**Published:** 1887-10

**Authors:** 


PHYSICIANS' MUTUAL BENEFIT ASSOCIATION OF TEXAS.—Dr. A. R. Kilpatrick was a member of this order, and held certificate No. 46. Assessment (No. 4) has been made on the members ($1 each) for the benefit fund provided by the constitution and by-laws, for his widow, and many of the members have promptly responded. We are sorry to say, however, that some are slow in coming up, and to date not over one-half have paid the assessment. In the case of the three preceding deaths (we are almost ashamed to say it), about ten per cent. of the members failed to pay in each case, and thus forfeited their membership. This will explain the discrepancy between the number of active members and the number of certificates issued. At the time of Dr. Kilpatrick's death, the number of active, paid-up members was 159; the certificates issued to that date were 201. On assessment and on annual dues thirty-eight in all have forfeited, and there have been four deaths. Mrs. K. should receive $159, less a few dollars for printing and postage, as provided by the by-laws. Since the date of the Doctor's death some three or four new member have joined; they, of course, are not subject to this assessment.

